# Double Iatrogenic Ethmoid-Orbito-Frontal Mucocele: The Role of Neuronavigation System in Revision of Endoscopic Sinus Surgery—A Case Report

**DOI:** 10.3390/reports9040347

**Published:** 2026-10-12

**Authors:** Chiara Rustichelli, Eleonora Ghessa, Alessandro Serrone, Guido Cavigioli, Giovanni Cavallo, Gian Luca Fadda

**Affiliations:** 1 Department of Otorhinolaryngology, University of Turin, “San Luigi Gonzaga” Hospital, Regione Gonzole 10, Orbassano, 10043 Turin, Italy; chiararustichelli75@gmail.com (C.R.); giovanni.cavallo@unito.it (G.C.); dott.fadda@gmail.com (G.L.F.); 2 Department of Surgical Sciences, Division of Otorhinolaryngology, University of Turin, 10126 Turin, Italy; eleonora.ghessa@unito.it (E.G.); guido.cavigioli@unito.it (G.C.)

**Keywords:** postoperative mucocele, frontal sinus, endoscopic sinus surgery, neuronavigation, case report

## Abstract

**Background and Clinical Significance**: Frontal sinus disease remains one of the most challenging conditions in rhinology due to its complex and variable anatomy and high tendency toward postoperative stenosis. Paranasal sinus mucoceles are uncommon, delayed complications following functional endoscopic sinus surgery (FESS), most often involving the frontal or anterior ethmoidal sinuses. They may arise from obstruction or scarring of natural drainage pathways, producing recurrent sinusitis or headache; **Case Presentation**: A 21-year-old male presented with persistent left-sided frontal headache and nasal obstruction after two previous FESS procedures. Radiologically, CT and MRI demonstrated chronic left-sided sinonasal inflammatory disease and two frontal-ethmoidal mucoceles. Revision endoscopic sinus surgery was performed with an image-guided (neuronavigation-assisted) technique, including Draf IIb frontal sinusotomy, mucocele marsupialization, and corrective septoplasty. The postoperative course was uneventful with rapid clinical improvement; **Conclusions**: Double postoperative mucoceles following ESS are rare. Neuronavigation-assisted revision surgery can be used for effective management in scarred, anatomically distorted sinus regions. However, this single case cannot establish superiority over conventional endoscopic guidance or demonstrate an effect on safety, efficacy, or recurrence.

## 1. Introduction and Clinical Significance

Frontal sinus diseases are still nowadays one of the most challenging issues in rhinology, due to their highly variable anatomy and the location of the natural ostium, which leads to difficult visualization during surgery. Therefore, its tendency to create stenosis is also well-known [1].

Additionally, in patients with a history of prior FESS procedures, surgeons may encounter altered anatomy, postoperative scarring, and an increased risk of complications, including postoperative lesions such as mucoceles [2].

Paranasal sinus mucoceles are benign, mucus-filled epithelium-lined cystic lesions that gradually expand due to drainage obstruction [3]. They affect people aged 40–60 years old, with no substantial difference between sexes [4].

The etiology of sinus mucoceles remains a subject of debate. They may develop as a result of chronic mucosal inflammation within obstructed sinuses, but are more frequently associated with prior trauma, benign tumors, or postoperative complications in which adhesions and scar tissue obstruct the operated paranasal sinus [5].

According to Molteni G. et al. [6], the obstruction of the frontal recess by an inflammatory postoperative scar process or tumor is indicated in 64% of the reported cases. In the remaining 36%, the etiology remains unknown.

Additionally, patients with Samter’s triad, comprising nasal polyposis, asthma, and aspirin sensitivity, are at increased risk as a result of chronic inflammation [7].

The term ‘mucopyocele’ (or ‘pyocele’) refers to an infected mucocele. It presents as an epithelium-lined cystic lesion filled with mucopurulent material and, like a mucocele, is thought to arise following direct trauma or prior surgery. These lesions typically enlarge progressively, extending into adjacent spaces and often causing erosion of the surrounding bony structures. They most commonly involve the frontoethmoidal region, likely due to its narrow anatomy and increased mucosal contact [8].

Radiologic differentiation with Computed Tomography (CT) and Magnetic Resonance Imaging (MRI) is crucial for the differential diagnosis of mucoceles. CT defines the degree of bone erosion and expansion, while MRI characterizes the lesion’s content and enhancement pattern, helping distinguish mucoceles from neoplasms or other cystic lesions. Mucoceles usually cause smooth bone expansion and remodeling, whereas neoplasms produce irregular bone destruction. Among benign lesions, the differential diagnosis of mucoceles includes mucopyoceles, mucous retention cysts, chronic sinusitis with osteitis, orbitofrontal cholesterol granuloma, dermoid and epidermoid cysts [9].

Frontal sinus mucocele may be asymptomatic in the very beginning, with a slow progression that may lead to a delay in diagnosis and treatment [10]. The most common symptoms reported are frontal headache and, in some cases, orbital involvement such as pain, proptosis, or diplopia [9,11].

The surgical management of chronic or refractory frontal sinusitis remains particularly challenging due to the sinus complex and variable anatomy, as well as its tendency toward postoperative scarring and stenosis [11]. Also, frontal sinus involvement is one of the most frequent causes of CRS recurrence after sinus surgery, mainly due to inadequate intraoperative management or an incomplete sinus opening [12].

When selecting the appropriate surgical approach for complex frontal sinus disease, we should consider two key factors: the patency of the ipsilateral frontal sinus outflow tract and the feasibility of endoscopic access to the mucocele. The endoscopic approach is the most popular nowadays, due to its safety and low rates of morbidity, recurrence, and complications; but if adequate frontal recess dissection cannot be achieved with an intranasal approach, a stepwise escalation to more invasive or external procedures may be required, including an external approach such as a Caldwell-Luc, osteoplastic frontal flap, or external fronto-ethmoidectomy [4].

The main clinical interest of this case lies not in the use of image-guided navigation itself, which is an established adjunct in complex revision endoscopic sinus surgery, but in the coexistence of two separate obstructive lesions within a severely altered ethmoid-frontal anatomy. The case illustrates the diagnostic challenge of distinguishing discrete postoperative mucoceles from persistent inflammatory disease and highlights the surgical importance of restoring adequate drainage pathways in a scarred and distorted frontal recess.

## 2. Case Presentation

A 21-year-old male presented in January 2026 with a persistent left frontal headache and nasal obstruction following two FESS procedures performed during 2025 (January and June) for chronic ethmoid-frontal rhinosinusitis. Because the previous surgeries were performed at another institution and the preoperative imaging and operative reports were unavailable, a definitive causal relationship could not be established. Therefore, the lesions are referred to as postoperative rather than iatrogenic mucoceles.

No relevant medical, family, or psychosocial history was reported. Nasal endoscopy revealed a patent left middle meatus but complete blockage of frontal and ethmoidal recesses, alongside septal deviation obstructing airflow. The clinical course and relevant surgical history are summarized in Table 1.

His symptoms were evaluated preoperatively using questionnaires: the 22-item Sino-Nasal Outcome Test (SNOT-22) was used to evaluate overall sinus-specific quality of life (QOL). This questionnaire contains 22 questions, each scored 0–5 (total score range 0–110), with higher scores representing more severe QOL impact. The SNOT-22 result was 40/110, compatible with a moderate impairment in quality of life. Perceived chemosensory dysfunction was assessed using the Chemosensory Complaint Score (CCS). This survey consists of questions pertaining to taste and smell. Scores range from 0–10 for the taste component and 0–6 for the smell component. Both scores were added to give the total chemosensory complaint score, with higher scores representing worse taste/smell: 2/16 (taste 2/10, smell 0/6); Sniffin’ identification test: 16/16.

He underwent routine laboratory testing: WBC 7.07 × 10^3^/µL, eosinophils 7.6% (absolute 0.53 × 10^3^/µL), total IgE 526 KU/L.

**Figure 1:**
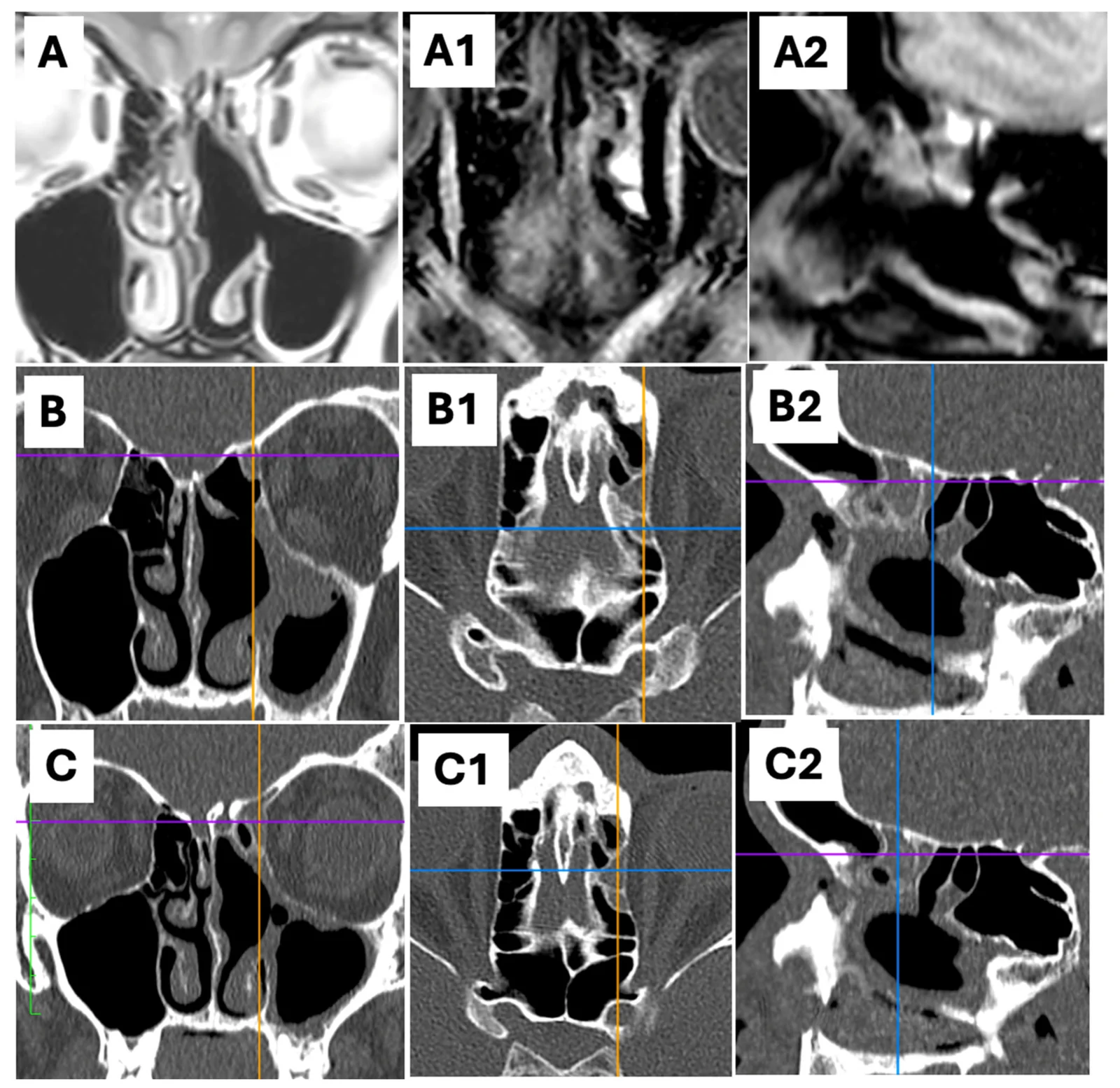
(**A**) Coronal, (**A1**) axial and (**A2**) sagittal T2W MRI, showing a hyperintense double left postoperative ethmoid-orbito-frontal mucocele. (**B**) Coronal, (**B1**) axial and (**B2**) sagittal CT scans show the mucocele involving the left anterior ethmoid sinus, and (**C**) Coronal, (**C1**) axial and (**C2**) sagittal CT scans show the second mucocele involving the frontal recess.

Preoperative CT scans demonstrated near-complete opacification of the left frontal sinus, with partial involvement of the contralateral side. MRI revealed extensive left-sided sinonasal inflammatory disease, characterized by diffuse mucosal thickening and two well-defined retention mucoceles resulting in complete opacification of the left frontal, maxillary, and ethmoidal sinuses. The first mucocele was centered in the agger nasi region (ethmoido-orbital, Figure 1B), while the second was identified in the ethmoido-orbito-frontal recess (Figure 1C). No significant diagnostic challenges were encountered during the evaluation.

Based on the clinical and radiological findings, the main differential diagnoses considered included mucocele/mucopyocele, chronic rhinosinusitis with osteitis, cholesterol granuloma, and other benign cystic or neoplastic lesions.

In February 2026, the patient underwent revision endoscopic sinus surgery under general anesthesia, using an image-guided neuronavigation system through the left nasal cavity. Given the distorted anatomy resulting from the previous surgical procedures, the image-guided neuronavigation system was used throughout the revision procedure to confirm the location of critical anatomical landmarks and to guide dissection through the scarred and hyperostotic sinonasal compartments. Navigation was particularly useful during identification and opening of the frontal recess and in defining the limits of the surgical field, helping to minimize the risk of injury to adjacent critical structures.

**Figure 2:**
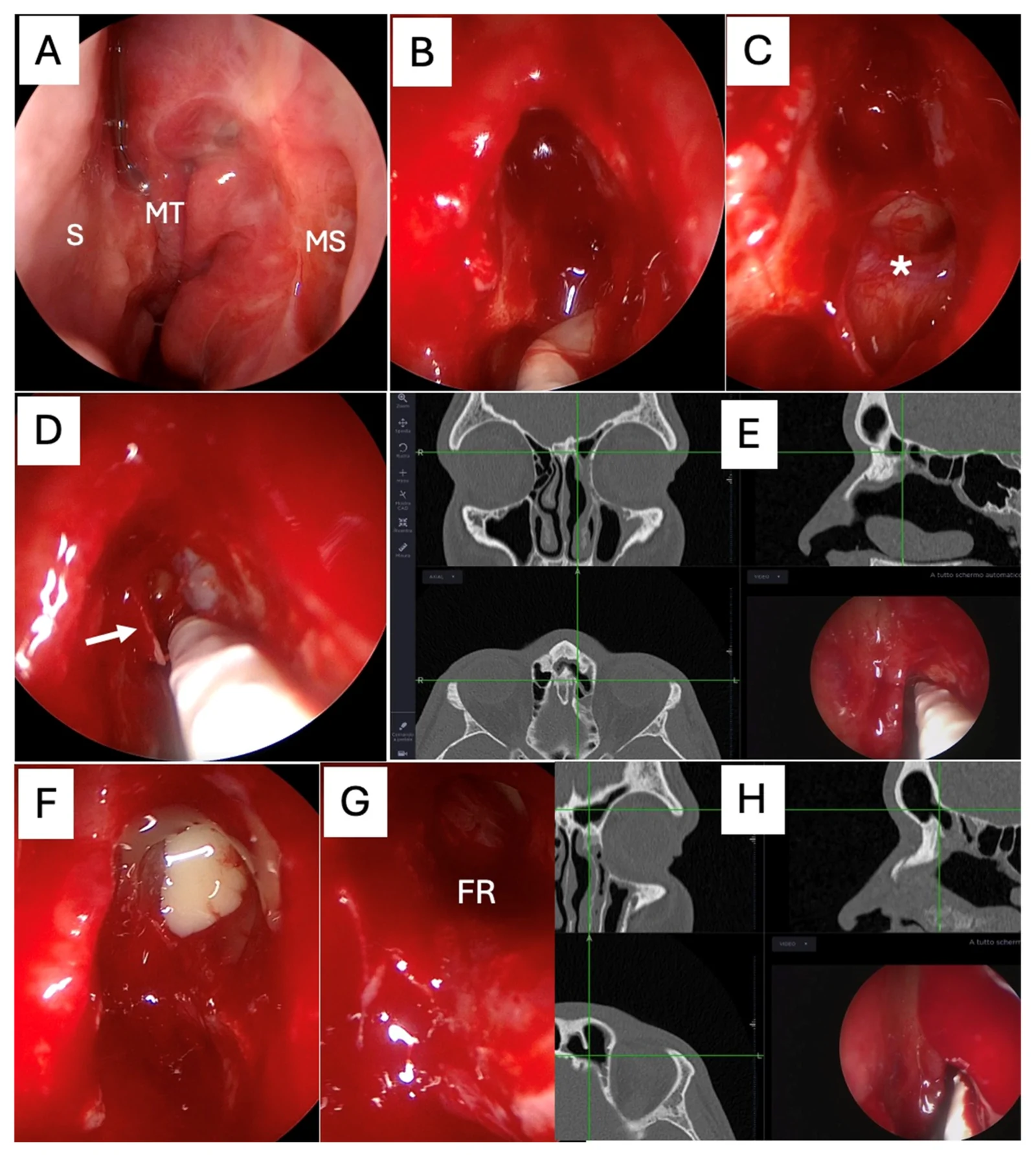
Intraoperative image during revision of endoscopic sinus surgery. (**A**) Left nasal cavity: scarred from previous surgery. (**B**,**C**) Marsupialization of the first mucocelic pouch in the anterior ethmoid sinus. Middle ethmoidal artery (asterisk). (**D**) Marsupialization of the second mucocelic pouch in the frontal recess. Medialized upper vertical uncinate process (white arrow). (**E**) Vision of the frontal recess with navigation system. (**F**) Mucopurulent material from the left frontal sinus in the second cystic cavity. (**G**) Draf IIb treatment on the left frontal sinus and (**H**) vision with navigation system. (S = septum; MT = middle turbinate; MS = maxillary sinus; FR = recess frontal).

Progressive posterior ethmoidectomy and sphenoidotomy were performed to restore anatomic visibility. Hyperostotic bone required drilling to access the frontal recess, where two ethmoido-orbito-frontal mucoceles were found. These two independent cystic cavities containing dense mucous material were identified and separately opened and marsupialized. The first one—in the anterior ethmoid/agger nasi region—had a mucous appearance, whereas the second one—identified more posteriorly and superiorly within the frontal recess—was sent for microbiological analysis since it had a mucopurulent appearance (Figure 2F). A Draf IIb frontal sinusotomy was completed to ensure wide drainage. Hemostasis was achieved using a Dessy coagulator, and a nasal pack was placed. No intraoperative or postoperative complications occurred (Figure 2).

The patient was discharged on the second postoperative day. Therapy included oral Ceftitoren 200 mg every 12 h for seven days, Betamethasone 2 mg daily for three days then tapered to 1 mg for three days, and isotonic nasal irrigations. The patient reported good adherence to the prescribed postoperative treatment, including antibiotic therapy and nasal saline irrigations, with no treatment-related adverse effects. No changes in the planned therapeutic intervention were required.

Given complete endoscopic drainage and restoration of frontal sinus ventilation, the postoperative prognosis was considered favorable, although endoscopic surveillance was recommended because of the history of previous FESS and postoperative scarring. At the 3-month postoperative follow-up, nasal endoscopy confirmed adequate frontal sinus ventilation and patency, with no evidence of recurring mucocele. The patient also reported improvement in frontal headache, nasal obstruction and nasal discharge. No postoperative complications were observed.

The mucopurulent material obtained intraoperatively from the second mucocele was sent for microbiological analysis, which yielded *Staphylococcus aureus*.

Histopathological examination of the mucosal tissue showed severe chronic inflammation with prominent eosinophilic infiltration, without evidence of neoplastic disease. Although histology did not represent an independent diagnostic criterion for mucocele, these findings, together with the characteristic radiological appearance and the intraoperative identification of two separate obstructed cavities containing mucous material, supported the final clinical diagnosis.

## 3. Discussion

Paranasal sinus mucoceles are benign, expansile, epithelial-lined lesions caused by obstruction of sinus drainage and, in 90% of cases, affect the frontoethmoidal complex followed by the maxillary sinus (10%) and rarely the sphenoid sinus without gender difference [13].

Mucocele formation secondary to FESS has not been frequently reported in the literature. This case highlights the diagnostic and therapeutic challenges of double frontal postoperative mucoceles following previous FESS. Mucoceles may develop months or years after surgery due to obstruction of mucosal drainage pathways from scarring, synechiae, or bony remodeling.

They usually grow slowly, but delayed diagnosis may result in extension into adjacent structures, including the orbit and anterior cranial fossa. Severe complications such as orbital involvement, intracranial extension, abscess formation, meningitis, fistula formation, and cerebrospinal fluid leakage have been described [14,15,16]. A previously reported case of bilateral frontal mucoceles with both orbital and intracranial extension further illustrates the potential for extensive disease when frontal sinus drainage is compromised [10]. These reports underline the importance of early diagnostic assessment and appropriate surgical management, particularly when frontal or ethmoidal mucoceles are associated with adjacent critical structures.

An important diagnostic consideration in this case was the distinction between true postoperative mucoceles and persistent or multiloculated inflammatory disease. In patients who have undergone previous endoscopic sinus surgery, postoperative scarring and anatomical distortion may result in retained secretions and chronic inflammatory opacification that can mimic a mucocele. In the present case, the diagnosis of two separate mucoceles was based on the combination of preoperative imaging, which demonstrated two anatomically distinct lesions, and intraoperative findings showing two independent obstructed cavities that were separately opened and marsupialized.

Histopathological examination demonstrated severe inflammatory changes without neoplastic features, supporting the inflammatory nature of the lesions but not serving as an independent diagnostic criterion for mucocele. The second mucocele, located in the frontal recess, contained purulent material, from which microbiological culture yielded *Staphylococcus aureus*, consistent with a frontal mucopyocele.

Endoscopic surgery by endonasal route is currently the treatment of choice in the marsupialization of mucoceles and establishment of drainage due to its low iatrogenicity and its excellent efficiency. Frontal sinus management is an important milestone in endoscopic sinus surgery, and if incomplete, it is one of the most frequent causes of recurrent CRS and complications. This is due to the difficult anatomy of this area, besides the incomplete dissection of the fronto-ethmoidal cells and the trauma caused to the mucosal lining with stripping [12]. According to that, we would underline the role of the surgical team’s expertise in approaching frontal sinus diseases.

In agreement with several authors [17,18], computer-assisted neuronavigation may provide valuable anatomical guidance during complex revision surgery, particularly in the frontal recess and frontal sinus. The use of image-guided navigation should not be considered the primary novel aspect of this case, as computer-assisted navigation is an established adjunct in revision endoscopic sinus surgery, particularly in patients with altered postoperative anatomy and disease involving the frontal sinus or structures adjacent to the orbit and skull base. Its value in the present case was therefore contextual rather than novel: although a single case cannot establish the superiority of neuronavigation over conventional endoscopic guidance, its use provided an additional layer of intraoperative anatomical orientation during dissection of the severely scarred and hyperostotic sinonasal compartments, facilitated correlation of the surgical field with the preoperative imaging, and supported identification of the frontal recess during the Draf IIb procedure. Thus, the educational value of this case lies primarily in the recognition and management of two distinct postoperative mucoceles in a markedly altered frontal-ethmoidal anatomy, rather than in the introduction of a novel navigation technique.

Draf IIb frontal sinusotomy involves resection of the frontal sinus floor from the lamina papyracea to the nasal septum, including the anterosuperior portion of the middle turbinate and the frontal sinus floor between the axilla of the middle turbinate and the nasal septum. The frontal recess was enlarged anteriorly and superiorly to create a wide, stable drainage pathway while preserving the skull base and orbital boundaries. This procedure provides a wide drainage route, minimizing recurrence risk, and it is indicated in inflammatory diseases and tumors and in revision surgery [19].

Postoperative monitoring is mandatory, since mucoceles may recur if drainage pathways re-close due to fibrosis. Regular endoscopic follow-up can help assess frontal sinus patency and detect possible recurrence. Nevertheless, the surgical outcome not only depends on a successful surgical technique, but also on the postoperative behavior of patients and their compliance with the medical indications after surgery [20]. Saline irrigations, topical medical treatment, and endoscopic cleaning or debridement may help maintain patency and support postoperative recovery.

The main strength of this case report is the description of a rare presentation of postoperative ethmoid-orbito-frontal mucocele and mucopyocele following previous endoscopic sinus surgery, supported by detailed radiological, endoscopic, and intraoperative documentation. Although image-guided neuronavigation is not novel in itself, its use in the present case provided additional anatomical guidance during revision surgery in a severely altered and scarred sinonasal environment, facilitating the identification of relevant anatomical landmarks and the completion of the frontal sinusotomy. This highlights its practical value as a useful adjunct to consider in similarly complex revision cases.

This report has also several limitations. First, as a single case report, the findings cannot be generalized and do not allow conclusions to be drawn regarding the superiority of image-guided navigation or its effect on recurrence or complication rates. Second, the postoperative follow-up was limited to three months, which does not allow assessment of long-term patency or recurrence. Finally, postoperative objective symptom scores were not available for comparison with the preoperative assessment. Further studies involving larger patient cohorts and longer follow-up are needed to better define the role of image-guided navigation in complex revision frontal sinus surgery.

## 4. Conclusions

Mucocele and mucopyocele formation is a rare postoperative complication of functional endoscopic sinus surgery, and the occurrence of postoperative mucoceles and mucopyoceles represents a rare and challenging clinical scenario. In revision surgery involving distorted anatomy and postoperative scarring, computer-assisted neuronavigation may provide valuable anatomical guidance, facilitating the safe identification of critical structures and restoration of frontal sinus ventilation. This case highlights the importance of meticulous surgical technique during primary FESS, appropriate postoperative surveillance, and consideration of image-guided navigation in complex revision procedures.

## Figures and Tables

**Table 1:** Timeline of the patient’s clinical course, including previous surgical interventions, presentation, revision surgery, and postoperative follow-up.

Date	Clinical Event
January 2025	First FESS performed for chronic ethmoid-frontal rhinosinusitis.
June 2025	Second FESS performed for chronic/recurrent ethmoid-frontal rhinosinusitis.
January 2026	Presentation with persistent left frontal headache and nasal obstruction; clinical, endoscopic, laboratory, and radiological assessment performed.
February 2026	Revision image-guided endoscopic sinus surgery with marsupialization of two mucoceles and Draf IIb frontal sinusotomy.
3 months postoperatively	Endoscopic follow-up demonstrated adequate frontal sinus ventilation and patency, with no evidence of mucocele recurrence; clinical symptoms improved.

## Data Availability

The original contributions presented in this study are included in the article. Further inquiries can be directed to the corresponding author.

## Abbreviations

The following abbreviations are used in this manuscript:

CCS	Chemosensory Complaint Score
CRS	Chronic Rhinosinusitis
CT	Computed Tomography
ESS	Endoscopic Sinus Surgery
FESS	Functional Endoscopic Sinus Surgery
IgE	Immunoglobulin E
MRI	Magnetic Resonance Imaging
SNOT-22	22-item Sino-Nasal Outcome Test
VAS	Visual Analogue Scale
WBC	White Blood Cell Count
